# Proteomic analysis showing the signaling pathways involved in the rhizome enlargement process in *Nelumbo nucifera*

**DOI:** 10.1186/s12864-019-6151-x

**Published:** 2019-10-22

**Authors:** Dingding Cao, Rebecca Njeri Damaris, Yue Zhang, Meihui Liu, Ming Li, Pingfang Yang

**Affiliations:** 1 Key Laboratory of Plant Germplasm Enhancement and Specialty Agriculture, Wuhan Botanical Garden, Chinese Academy of Sciences, Wuhan, 430074 China; 2 University of Chinese Academy of Sciences, Beijing, 100049 China; 3 State Key Laboratory of Biocatalysis and Enzyme Engineering, School of Life Sciences, Hubei University, Wuhan, 430062 China

**Keywords:** *N. nucifera*, Rhizome enlargement, Proteomics, Morphology, Signaling

## Abstract

**Background:**

Rhizome is the storage underground stem of lotus (*Nelumbo nucifera*), which is enlarged before winter season and could be used for asexual propagation. In addition, the enlarged rhizome is a nutritional vegetable with abundant starch, proteins, and vitamins. Enlargement of lotus rhizome is not only significance for itself to survive from the cold winter, but also important for its economic value.

**Results:**

To explore the mechanism underlying its enlargement, integrative analyses of morphology, physiology and proteomics were conducted on the rhizome at stolon, middle, and enlarged stages. Morphological observation and physiological analyses showed that rhizomes were gradually enlarged during this process, in which the starch accumulation was also initiated. Quantitative proteomic analysis on the rhizomes at these three stages identified 302 stage-specific proteins (SSPs) and 172 differently expressed proteins (DEPs), based on which GO and KEGG enrichment analyses were conducted. The results indicated that light and auxin signal might be transduced through secondary messenger Ca^2+^, and play important roles in lotus rhizome enlargement.

**Conclusion:**

These results will provide new insights into understanding the mechanism of lotus rhizome enlargement. Meanwhile, some candidate genes might be useful for further studies on this process, as well as breeding of rhizome lotus.

## Background

Nelumbo, with a popular name lotus, is an aquatic herbage belonging to the *Nelumbonaceae* family. It comprises two extant species: Asia lotus (*N. nucifera* Gaertn.) and America lotus (*N. lutea* (Wild.) Pers). Asia lotus mainly distributes in Asia and Northern Australia, while America lotus is only discovered in North America and north of South America [1]. There are two ecotypes of *N. nucifera* named as temperate and tropical lotus. The underground stem of tropical lotus shows no significant enlargement, while that of temperate ecotype is enlarged into rhizome at the end of its growth [2], which is similar to the tuber of potato (*Solanum tuberosum*), sweet potato (*Ipomoea batatas*) [3], and bulb of onion (*Allium cepa*) [4]. Being a storage tissue, rhizome is not only used as vegetative reproductive tissue in agriculture for lotus production, but also as an important and nutritional vegetable in Southeast Asia for its richness in nutrients including starch, proteins, and vitamins [5, 6]. The enlargement of rhizome could ensure lotus survival from the cold winter. Meanwhile, it also determines the yield of rhizome in agriculture production.

In general, the development of lotus rhizome could be divided into four stages: stolon stage, initial stage of expansion, middle stage and late stage of expansion [7, 8]. Several studies, including transcriptomics, genomics, and physiology, have been carried out in lotus focusing on this process, and showed that short day lighting and low temperature could promote rhizome formation [9–12]. Additionally, genes and proteins related to phytohormone, photoperiod, starch synthesis and flowering are indicated to be involved in the lotus rhizome formation [13–16]. Current understanding on features of lotus rhizome development indicate the possibility of lotus being another model plant in studying the regulation of storage organ formation in addition to potato [1, 7, 8].

Although mechanism of tuberization has been extensively studied in potato [17], there are obvious differences between potato and lotus rhizome. Potato has two types of stem, aerial stem and tuber, with the initiation of tuber formation being dependent on inducing signal from the environment. However, lotus only has subterraneous stem, which grows normally at the very beginning until the induction of enlargement. Although several studies at genomics, transcriptomics and physiology have been conducted focusing on this topic, there is still insufficient explanation, especially at protein level, on the enlargement of lotus rhizome. Proteomics have been widely applied in the studies on storage organs, such as the horsetail (*Equisetum hyemale*) underground stem, potato tuber and lotus rhizome, the ancient vascular plant and crop plant [18–22]. In the present study, we conducted a quantitative proteomic study on lotus rhizome at stolon stage, middle stage, and rhizome stage (hereafter, named as S1, S2 and S3 respectively). During these three stages, the enlargement of rhizome is initiated and largely completed. Meanwhile, the proteomic data were compared with our previous transcriptomic data, which might highlight the involvement of certain critical genes and the corresponding pathways in the development of lotus rhizome.

## Methods

### Plant materials

Temperate lotus cultivar ‘WZ II’ was used in this study, which has typical rhizome enlargement phenotype. This cultivar was bred and named by Prof. Xuemin Ni (Retired from Wuhan Botanical Garden, Chinese Academy of Sciences). It is popularly cultivated in agriculture production, and could be easily obtained in the market. They were cultivated in Wuhan Botanical Garden (N30°32′48.33″, E114°25′3.98″) with normal water and fertilizer management. Here ‘WZ II’ rhizome samples were manually collected in the second half of August at stolon stage (S1), middle stage (S2), and rhizome stage (S3). The rhizome enlargement index was measured following the method described by Masuda et al. [23]. Three biological replicates were used for the measurement, with each replicate containing three rhizomes. After measurement, the same set of samples were immediately frozen in liquid nitrogen and kept at − 80 °C until used for protein and RNA extraction.

### Microscopy observation and measurement of soluble sugar and starch

For microscopy observation, the rhizome samples at S1, S2 and S3 were collected and immediately fixed in formalin-acetic acid-alcohol for at least 1 d. Fixed samples were vacuum-dried for 1 h to remove the air inside samples, and then dehydrated with 30–100% gradient ethanol. Thereafter, the chloroform was used to replace the ethanol inside samples. After soaking into xylene solution containing paraffin, samples were embedded into pure paraffin. The embedded samples were cut into 6–10 μm thickness slices using LEICA2150 rotary microtome (Leica, German). Olympus BX-61 upright metallurgical microscope (Olympus, Japan) was employed to observe histological characteristics of the slices after PAS (Periodic Acid-Schiff stain) staining. Determination of total soluble sugar and starch contents were carried out according to the anthrone colorimetric method [24].

### Protein extraction and trypsin digestion

Proteins were extracted from the rhizome by phenol extraction method as previously described [25]. Protein concentration was measured using the Bradford assay with bovine serum albumin as the standard [26]. For trypsin digestion, the protein pellet was re-suspended in lysis buffer (7 M urea, 2 M thiourea, 4% CHAPS, 1 mM DTT and 1 mM PMSF). After dissolving, the concentration of protein was measured, and subsequently incubated in 10 mM DTT for 1 h at 56 °C. After cooling down to room temperature, the sample was alkylated by incubating in 40 mM iodoacetamide for 30 min in the dark. Subsequently, the sample was diluted with seven to eight volumes of distilled deionized water, then trypsin was added to the protein sample in a 1:50 ratio (trypsin/protein: w/w). The protein sample was digested by incubating at 37 °C on a rocking shaker overnight.

### Nano LC-MS/MS analysis

The digested peptides from total protein were re-suspended in 5% ACN and 0.1% formic acid, and then separately loaded into a nano LC instrument nano ACQuity (Waters, USA) with the cHiPLC trap (200 μm × 500 μm ChromXP C18-CL, 3 um, 300 Å) and washed for 15 min with a flow rate of 2 μL/min. Then, an elution gradient of 7–52% ACN (0.1% formic acid) for 44 min with flow rate of 300 nL/min was used on a nano cHiPLC column (75 μm × 15 cm ChromXP C18-CL, 3 μm, 300 Å). The MS analysis was performed on a Nanospray III source and a TripleTOF 5600 plus (AB SCIEX, USA) mass spectrometer in an information-dependent acquisition mode (27). MS spectra were acquired across the mass range of 350–1500 m/v in high-resolution mode (> 25,000) using 250 ms accumulation time per spectrum. A maximum of 30 precursors per cycle was chosen for fragmentation from each MS spectrum, with 50 ms minimum accumulation time for each precursor and dynamic exclusion for 12 s. Tandem mass spectra were recorded in high-resolution mode (resolution > 25,000) with rolling collision energy.

### Data acquisition and peptide analysis

The raw mass spectrometry MS/MS spectra search was processed using MaxQuant-associated Andromeda search engine [27], and a lotus peptide database (14,833 peptides) was used from LOTUS-DB (http://lotus-db.wbgcas.cn/) [28, 29]. Initial maximum precursor and fragment mass deviations were set to 6 ppm and 0.5 Da, respectively. Variable modification (methionine oxidation and N-terminal acetylation) and fixed modification (cysteine carbamidomethylation) were set for the search, and trypsin with a maximum of two missing fragmentation were chosen for searching. The minimum peptide length was set to 7 amino acids and the FDR (false discovery rate) for peptide and protein identification was set at 0.01. The precursor ion mass accuracy was improved using the time- and mass-dependent recalibration option software. The FDR was controlled at various levels by using a target-decoy search strategy, which integrates multiple peptide parameters such as length, charge, number of modifications and the identification score into a single quality that acts as the statistical evidence on the quality of each single peptide spectrum match [30]. The frequently observed laboratory contaminants were removed and the protein identification was considered valid only when at least 2 matched peptides and 1 unique peptide were present.

### Analysis of differentially abundant proteins using acquired mass spectrometry data

The software Perseus (version 1.4.1.3) (http://141.61.102.17/perseus_doku/doku.php?id=start) was used to compare the peak intensities across the whole set of measurements to obtain quantitative data for all of the peptides in the sample. The LFQ (label-free quantitation) was performed through MS/MS signal intensity. The integrated peak intensities (ion currents) of parent peptides were integrated and used to compare protein expression levels between samples using Andromeda algorithm. Protein quantification and calculation of statistical significance was carried out using Student’s t-test and error correction (*p* < 0.05) using the Benjamini–Hochberg method. For the heatmap, the sample LFQ values (Ls) were normalized for each protein (l) as follows: norm. l = [l – min (Ls)] / [max (Ls) – min (Ls)], with l ∈ Ls. All the proteins that showed a fold-change of at least 1.2 and satisfied *p* < 0.05 were considered to be differentially expressed. The database of Kyoto Encyclopedia of Genes and Genomes (KEGG), Gene Ontology (GO) were performed to categorize and group candidate proteins. The GO and pathways with a corrected *p* < 0.05 were considered as significant.

### Transcriptome data acquisition

RNA isolation, library construction, and sequencing for transcriptome was conducted as described by Yang et al. [7], and the samples of three stages were selected as rhizome formation stage, named T1, T2, and T3, corresponding to stage S1, S2, and S3 in the present proteome study.

### RNA extraction and quantitative reverse transcription polymerase chain reaction

Total RNA of S1, S2 and S3 samples were extracted using an RNA reagent (OminiPlant RNA Kit, CWBIO, China). Primers were designed with the Primer 3.0 software (http://biotools.umassmed.edu/bioapps/primer3_www.cgi). qRT-PCR reactions were performed in the CFX Connect (BIO-RAD) using the SYBR Green Master Mix (BioRad, http://www.bio-rad.com/), and amplified with 1 μL of cDNA template, 5 μL of 2 × SYBR Green Master Mix, and10 μM of each primer, to a final volume of 10 μL. The amplification program consisted of 1 cycle of 95 °C for 10 s, followed by 40 cycles of 95 °C for 15 s and 60 °C for 30 s. Fluorescent products were detected in the last step of each cycle. Melting curve analysis was performed at the end of 40 cycles to ensure proper amplification of target fragments. Each gene was performed in three technical replicates. The relative expression of each gene was normalized by comparison with the expression of lotus β-actin (*NNU_24864*) and analyzed using the 2^-∆CT^ Method .

## Results

### Morphological and physiological characterization of rhizome development

**Fig. 1:**
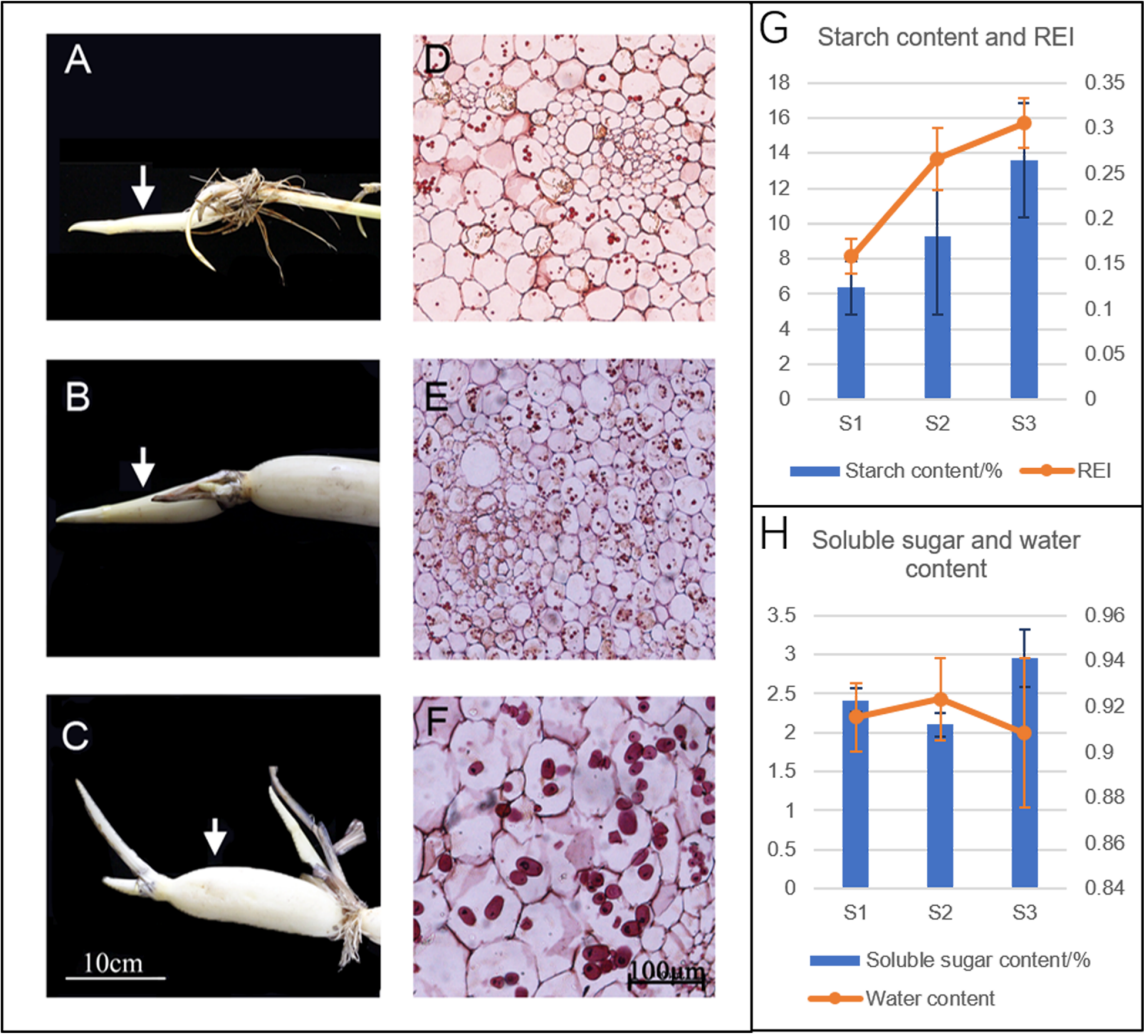
Morphological and physiological traits of lotus rhizome at S1 to S3 stages. **a-c** shows the growth and changes of lotus rhizome size at stage S1, S2 and S3. Bars are 10 cm; and arrows indicate the tissues collected for proteome analysis. **d-f** represent the PAS staining transverse sections of lotus rhizome at S1, S2 and S3. Bars indicate 100 μm. **g** shows the starch content (dry weight) and rhizome enlargement index (REI); **h** shows soluble sugar (dry weight) and water content. Data are means ± se (*n* = 3)

As an important storage organ in lotus, rhizome expansion could determine its final yield and quality of the product. However, its development is a complex process, which could be divided into four stages based on its morphological changes. According to our previous study, the expansion of rhizome is almost over at S3, after which it goes into the starch accumulation stage [6]. Since we are focusing on the enlargement, only the rhizomes at S1, S2 and S3 stages were used in this study. During these stages, lotus underground stem develops from the thin stolon into the expanded rhizome, with the expansion being initiated at the time between S1 and S2 (Fig. 1a-c). This could be easily judged by the increase of rhizome enlargement index (REI) from 0.09 to 0.35 (Fig. 1g), which is consistent with previous studies [7, 8, 23, 31]. In order to know if the enlargement is because of the cell division or growth or both, the transverse sections of the rhizome from the three stages were prepared and observed through microscope. Both the number and size of parenchymal cells were increased over the three stages (Fig. 1d-f). Specifically, statistics analysis suggested that cell number significantly increased from S1 to S2, and cell size significantly increased from S2 to S3 (Fig. 1d-f; Table 1). Furthermore, we also found the increase of starch content as well as the size of starch granule during this process (Fig. 1d-f, g). The contents of soluble sugar were constant between S1 and S2, with a little increase at S3, which is contrast to the water content showing a slight decrease at S3 (Fig. 1h). The morphological and physiological results were generally consistent with previous studies [31, 32].

**Table 1:** The cell number, cell volume, and starch content of lotus rhizome at S1, S2 and S3

Sample name	Cell number (300 × 300 μm^2^)	Single cell size (μm^2^)	Starch grain number per cell
S1	61.33 ± 2.08 ^b^	2536 ± 365 ^b^	mainly ≤3
S2	85.67 ± 6.66 ^c^	1585 ± 273 ^a^	mainly ≥5
S3	21.33 ± 3.21 ^a^	6434 ± 1172 ^c^	mainly 2–3

The different letters (a,b,c) indicate signifcant changes according to one-way ANOVA using Duncan’s test (*p* < 0.05)

### Profiling the proteome and its dynamic changes during rhizome development

**Fig. 2:**
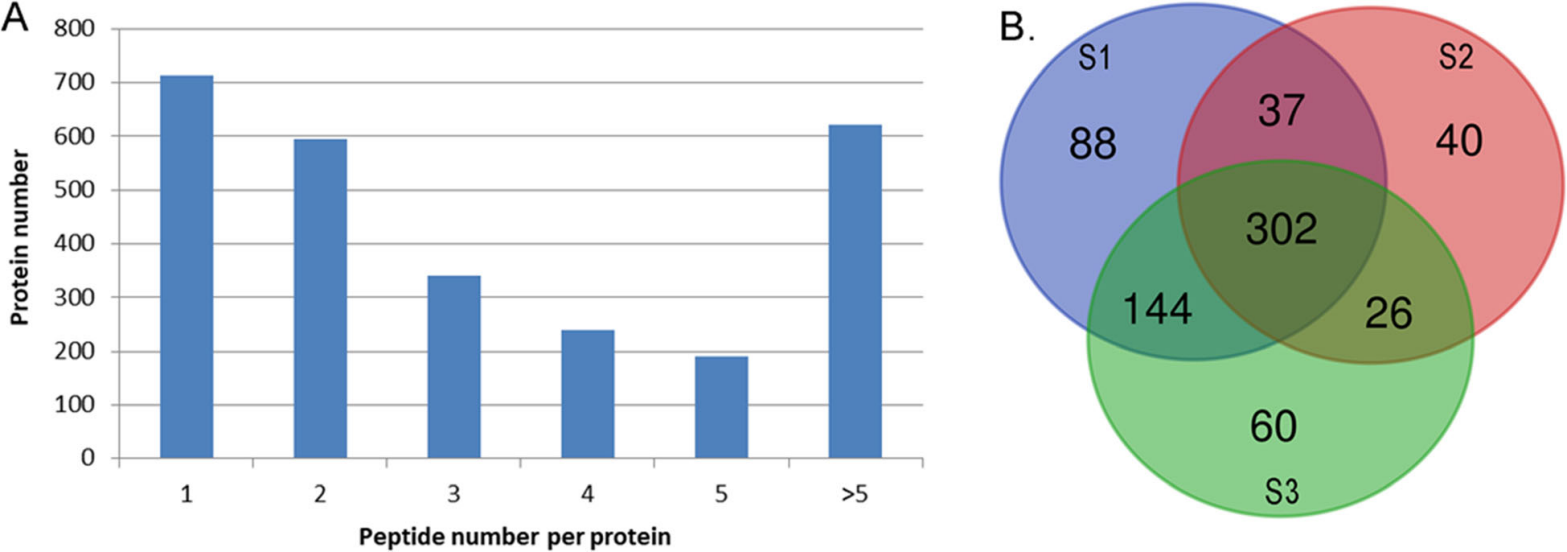
Overview of the proteome profile. **a** Distribution of the detected peptides among the identified proteins. **b** Venn diagram showing the stage-specific and overlapped proteins that were quantified among the three stages

To further understand the mechanism underlying its enlargement, label-free quantitative proteomics analysis was applied on the rhizome during this process. Proteins were extracted from the samples at S1, S2 and S3 stages separately, and then subjected to digestion and Mass spectrometry (MS) analysis as described in M&M. After filtering of the raw MS data, a total of 7936 unique peptides corresponding to 2712 proteins were identified from the rhizome (Fig. 2a; Additional file 1: Table S1). Expressions of the genes encoding all the identified proteins have been previously detected at mRNA level in our transcriptomic study (Additional file 1: Table S1). Among these proteins, 696 could be quantified. Further analysis showed that there were 570, 405 and 532 proteins detected in S1, S2 and S3, respectively, with 302 overlapping proteins at all three stages (Fig. 2b). Except for 172 of the overlapped proteins, all the other proteins were differentially expressed based on the threshold of 1.2-fold change in abundance (*p* < 0.05), including the stage-specific proteins (SSPs) for S1, S2 and S3 being 88, 40 and 60, respectively (Fig. 2b).

**Fig. 3:**
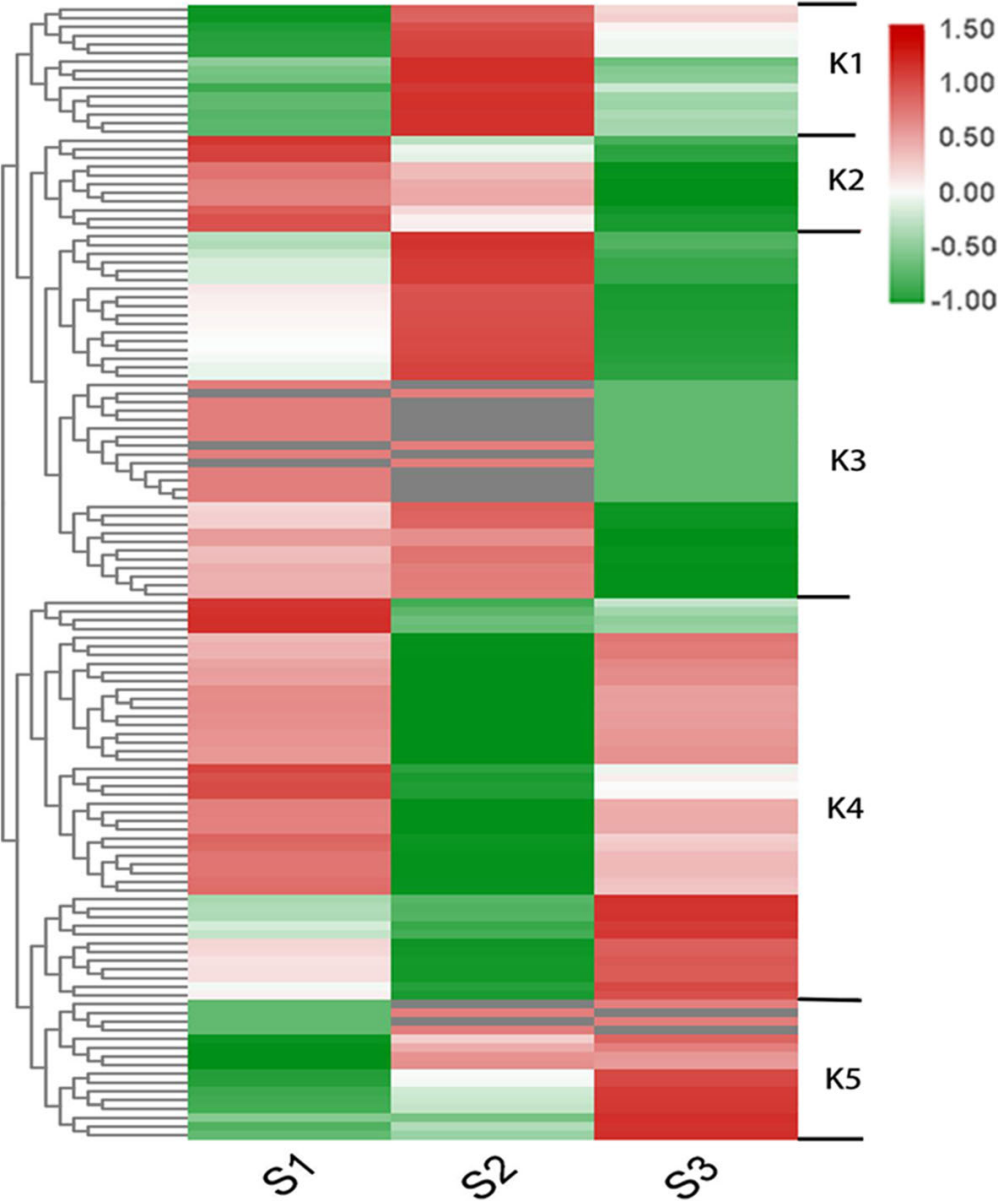
Heatmap showing the expressional patterns of the changed proteins. Based on the protein’s dynamic patterns, 6 clusters, named as K1-K6, were determined

To further understand the expressional styles of the DEPs, cluster analysis was conducted (Fig. 3). The DEPs could be divided into five groups (K1- K5) (Fig. 3, Additional file 2: Table S2). In K1, proteins showed a sharp up-regulation between S1 and S2, and then slowly down-regulated between S2 and S3. Proteins in cluster K2 were gradually decreased. Proteins in K3 were slowly up-regulated between S1 and S2, and then sharply down-regulated between S2 and S3. In contrast, K4 showed a down-regulated then up-regulated expressional style. In contrast to K2, proteins in K5 were gradually increased.

### GO and KEGG analysis

**Fig. 4:**
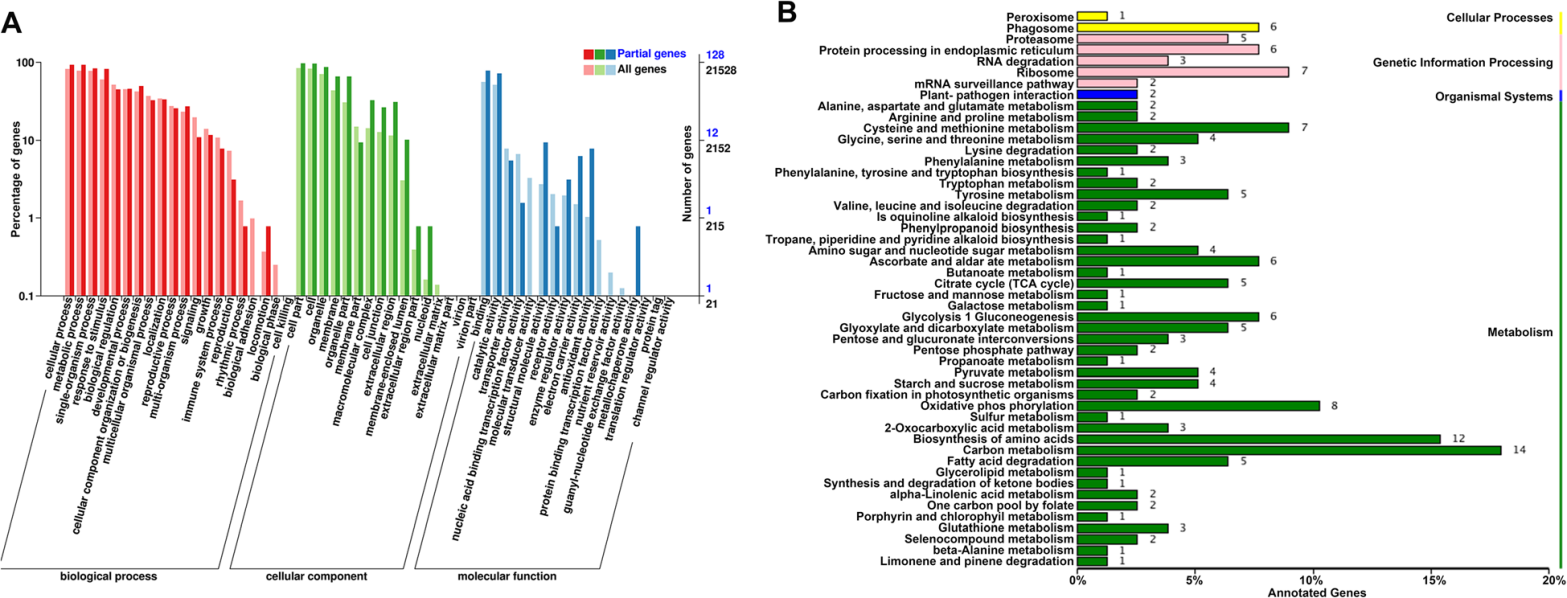
GO and KEGG analyses of the quantified proteins. **a** GO analysis of all the identified proteins under the background of all annotated genes. Y-axis on the right side represents the number of genes. The digital numbers in black represent the number of whole annotated genes, and those in blue are the numbers of the identified proteins. **b** KEGG enrichment analysis

**Fig. 5:**
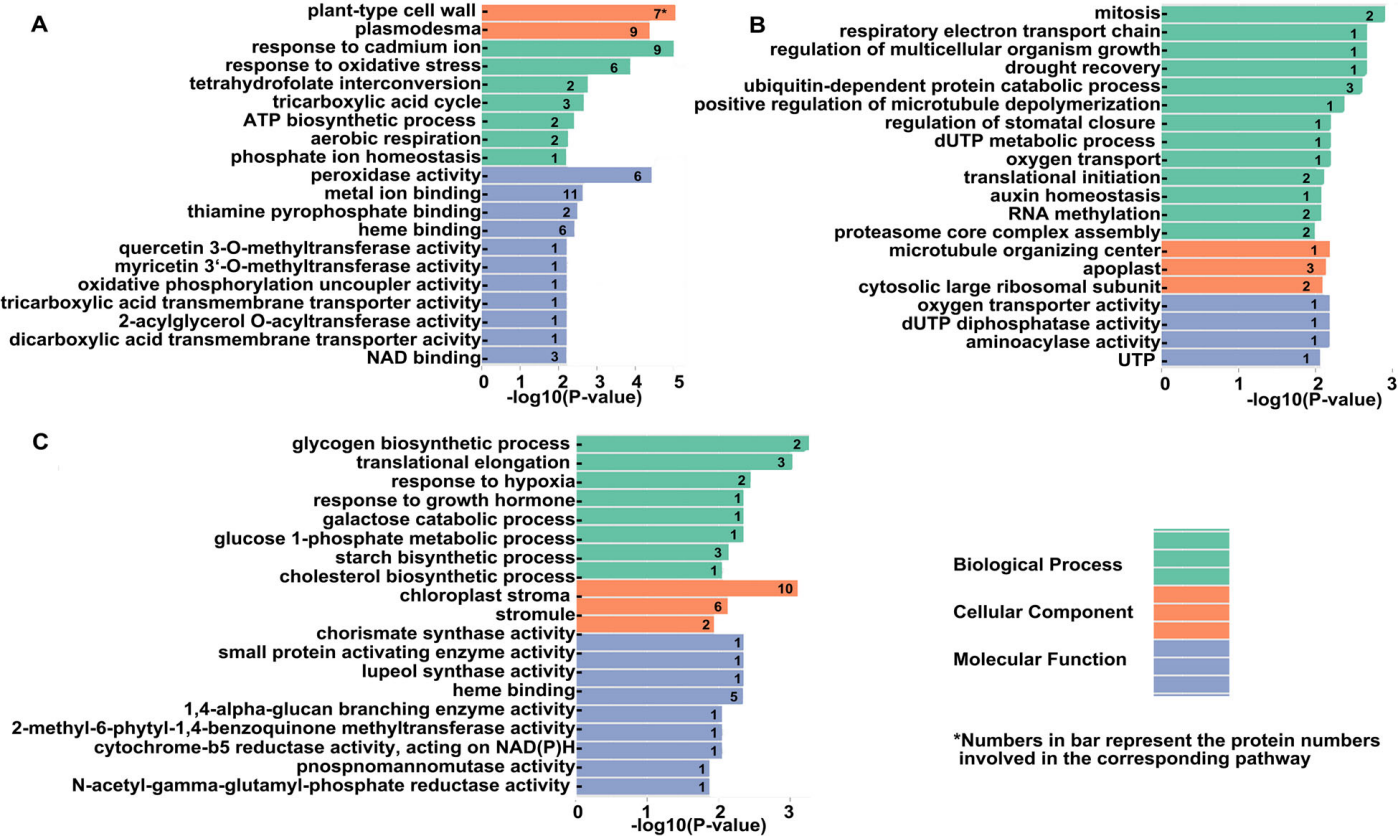
GO analysis of the stage-specific proteins. **a** for S1, **b** for S2, **c** for S3

To further understand the functions of 130 DEPs and 188 SSPs, GO and KEGG analyses were performed. The results showed that they could be assigned to 20 biological processes, 15 cell components, and 14 molecular function groups (Fig. 4a). Further analysis showed that the proteins involved in metabolism (especially amino acids and sugar metabolism), genetic information processing and some cellular processes were enriched (Fig. 4b). Since this is a dynamic analysis, we paid special attention to the stage specific proteins. The enriched GO terms were quite different among the three stages (Fig. 4). The oxidative and metal ion response related terms were enriched at S1 (Fig. 5a); the cell growth and division related terms were enriched at S2 (Fig. 5b); and the starch biosynthesis related terms were obviously enriched at S3 (Fig. 5c). The enrichment of cell growth and division proteins at S2, and starch biosynthesis proteins at S3 was consistent with the microscope observation and physiological data. These stage-specific enriched GO terms might be associated to the different growth features of each stage. Besides, KEGG pathway analysis showed that 105 items were enriched, with over 33% of the proteins being significantly enriched in either carbon metabolism or biosynthesis of amino acids (Additional file 2: Table S3).

### Comparison between proteomic and transcriptomic data and qRT-PCR validation

**Fig. 6:**
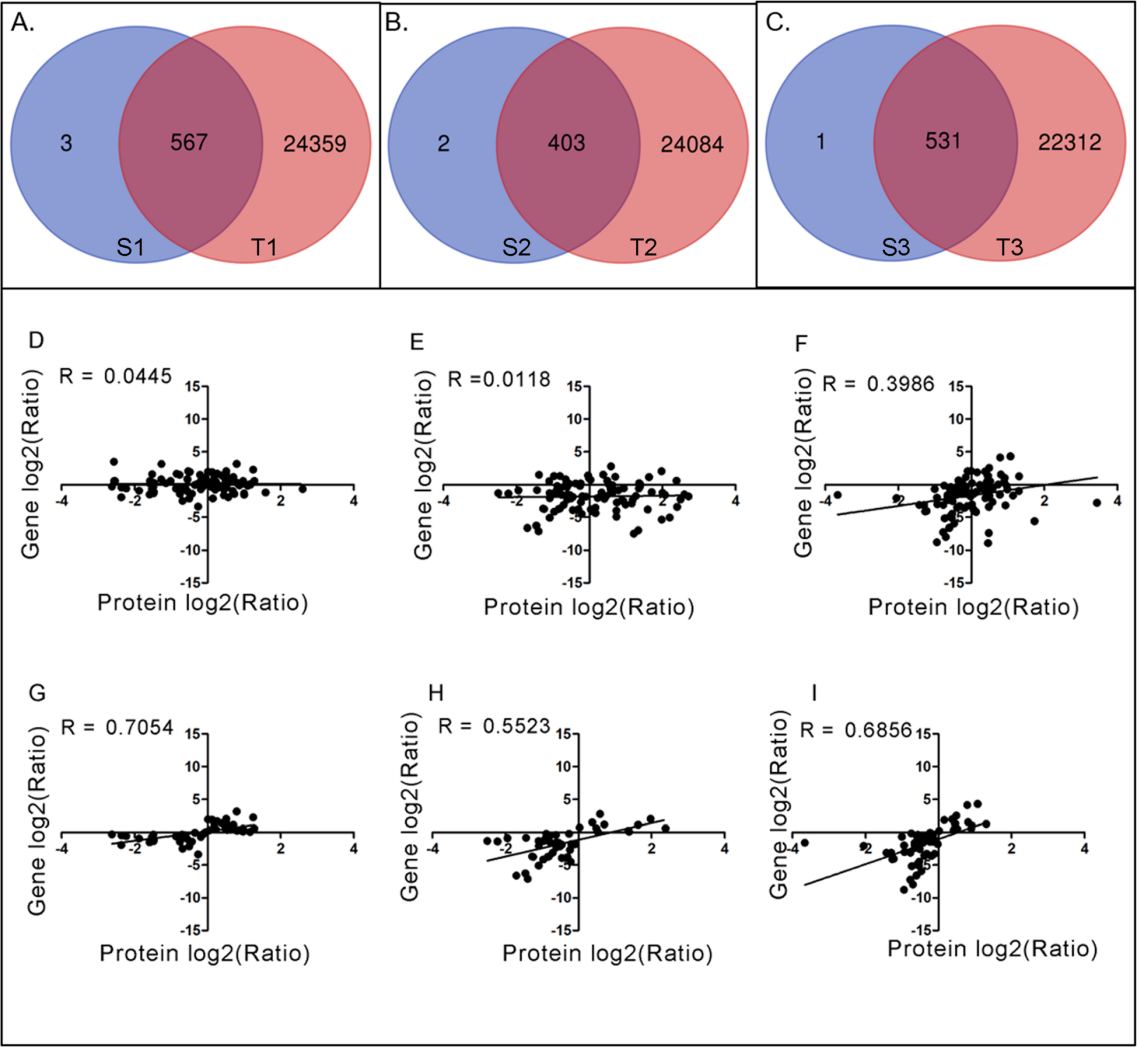
Correlation analysis between protein and mRNA data. **a**, **b**, and **c**, shows the comparison of transcriptome and proteome data at stolon stage, middle stage and rhizome stages (T1, T2, and T3 represent data from transcriptome and S1, S2, and S3 represent data from proteome). **d-f** shows the Pearson correlation coefficients of DEPs in three comparison groups. **g-i** shows the R value of DEPs with the same trends of expression. **d** and **g** represent S1 vs. S2 pair. E and H represent S2 vs. S3 pair. F and I represent S1 vs. S3 pair. R is the correlation coefficient

**Fig. 7:**
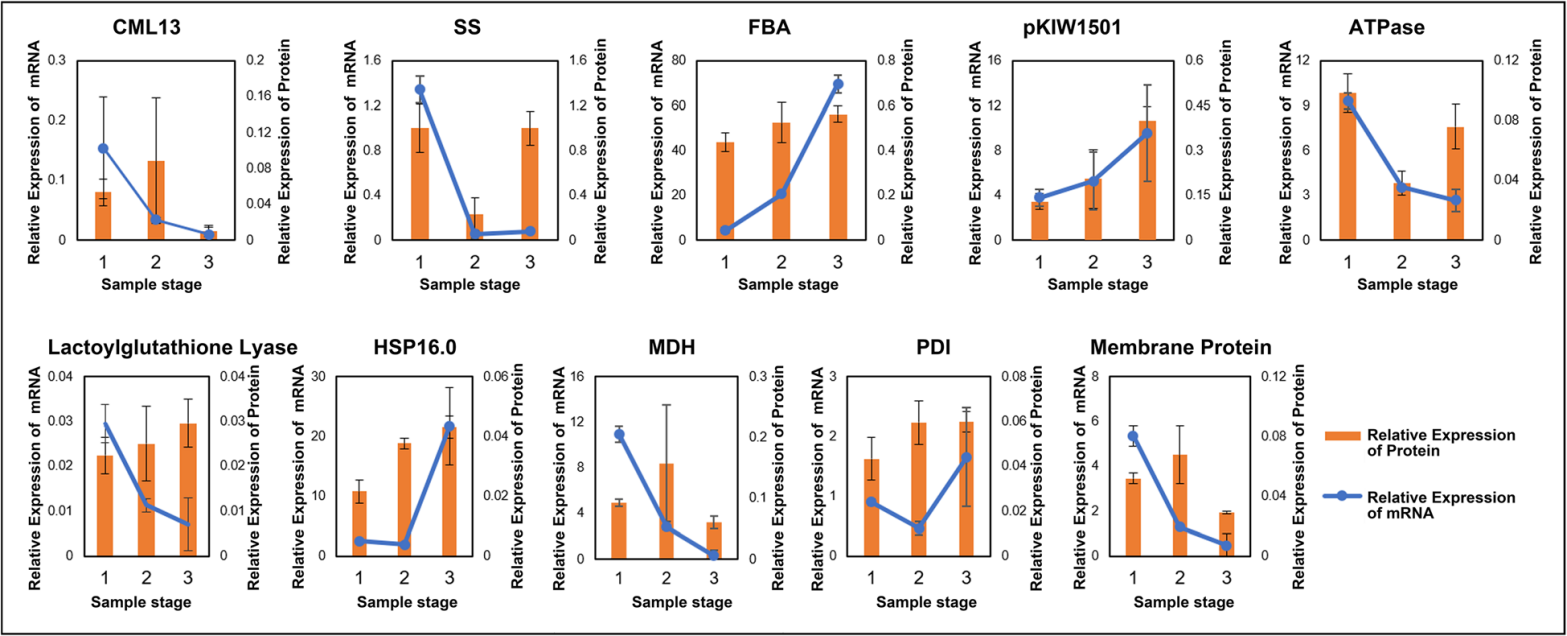
Validation of the proteome data by qRT-PCR. The data for protein and mRNA levels were integrated into one panel for each gene, with the histogram showing the protein level and the line showing the mRNA level. The x-axis is the three stages of sampling. Data are means ± se from three replicates

To further know the regulation on the genes encoding the quantified proteins, we compared our proteomic data with previous transcriptomic data [7]. Although all the identified proteins could find their corresponding mRNA in the transcriptomic data, some proteins did not have their corresponding mRNAs at the same time point. There were 567, 403, and 531 overlapped genes between transcriptome and proteome at stages of S1, S2 and S3, respectively (Fig. 6a-c). Meanwhile, all 130 DEPs could find their corresponding mRNA data in the transcriptomic analysis. The corresponding Spearman correlation coefficient for proteome and transcriptome in the pairs of S2 vs S1, S3 vs S2, and S3 vs S1 (R, Spearman) were 0.0445, 0.0118, and 0.3986, respectively (Fig. 6d-f). Nevertheless, when selecting the genes with the same trend of expression at both mRNA and proteins levels, the correlation coefficient increased to 0.7054, 0.5523 and 0.6856, respectively (Fig. 6g-i). To validate the relationship between the proteomic and transcriptomic data, 10 genes were selected for qRT-PCR analysis (Fig. 7). The results were generally consistent with the comparisons between transcriptomic and proteomic data (Fig. 7).

### Signaling and function important proteins

Among all the identified proteins, there were many involved in different signaling pathways, such as light, calcium, phytohormone, among others. There were 11 light signaling and circadian related proteins identified, including PHYA, FYPP, HY5, EFL4, FRIGIDA and 3 subunits of COP9 signalosome complex (Table 2). Both PHYA and FYPP had high level at S1, and HY5 had high level at S2. Three subunits of COP9 signalosome had high levels at either S2 or S3. Both EFL4 and FRIGID had high levels at S2. A total of 34 calcium signaling related proteins were identified, which included calcium transporter, calmodulin, annexin and calcium-dependent kinase (Table 2). Generally, most of these calcium proteins mainly accumulated at S1 either specifically or preferentially. In detail, the calcium transporters, annexins and calcium-dependent kinase mainly accumulated at S1 and S3, whereas, the calcium binding proteins were accumulated throughout all the 3 stages with higher level at S1 and S2 (Table 2). Impressively, 22 proteins involved in auxin signaling were identified, most of which had highest levels at either S1 or S2. A total of eight 14–3-3-like proteins were also identified in this study, with most of them having high levels at S1 (Table 2).

**Table 2:** The important signaling proteins that were identified

Protein ID	Function description	Relative expression level^a^		
		S1	S2	S3
Light signaling				
NNU_05425-RA	CSN4: COP9 signalosome complex subunit 4	1	N/A	N/A
NNU_15063-RA	CSN5A: COP9 signalosome complex subunit 5a	N/A	1	N/A
NNU_08779-RA	CSN1: COP9 signalosome complex subunit 1	N/A	N/A	1
NNU_09851-RA	PHYA: Phytochrome A	1	N/A	0.85
NNU_22076-RA	NDPK2: Nucleoside diphosphate kinase 2C chloroplastic	N/A	1	N/A
NNU_14358-RA	Similar to Nucleoside diphosphate kinase B	1	1.19	0.71
NNU_17592-RA	FYPP: Phytochrome-associated serine/threonine protein phosphatase	1	N/A	N/A
NNU_20168-RA	Similar to At1g80670: Rae1-like protein; may be involved in the formation of a CUL4-based E3 ubiquitin ligase	N/A	1	N/A
NNU_26357-RA	HY5: Transcription factor HY5	N/A	1	N/A
NNU_11842-RA	Similar to EFL4: Protein ELF4-LIKE 4	N/A	1	N/A
NNU_16002-RA	Similar to FRI: Protein FRIGIDA	N/A	1	N/A
NNU_13682-RA				
Calcium signaling				
NNU_15726-RA	Similar to VCX1: Vacuolar calcium ion transporter	N/A	N/A	1
NNU_15143-RA	Similar to ECA4: Calcium-transporting ATPase 2C endoplasmic reticulum-type	1	N/A	1.16
NNU_12400-RA				
NNU_05804-RA	Similar to Os12g0586600: Calcium-transporting ATPase 2C plasma membrane-type	1	N/A	N/A
NNU_04273-RA	Similar to CHERP: Calcium homeostasis endoplasmic reticulum protein	1	N/A	N/A
NNU_12646-RA	Similar to cbpP: Calcium-binding protein P	1	1.46	N/A
NNU_17533-RA	Similar to CALM1: Calmodulin	1	1.11	N/A
NNU_10822-RA	Similar to CAM-1: Calmodulin-1/11/16	1	1.22	1.58
NNU_20726-RA	Similar to CAM72: Calmodulin-2	N/A	N/A	1
NNU_01778-RA	Similar to CML6: Calmodulin-like protein 6	1	1.31	1.07
NNU_23435-RA	Similar to CML11: Calmodulin-like protein 11	N/A	N/A	1
NNU_06675-RA	Similar to CML13: Probable calcium-binding protein CML13	1	1.14	0.19
NNU_08869-RA				
NNU_05884-RA	Similar to Calreticulin	1	1.06	0.8
NNU_02729-RA	Similar to CRT3: Calreticulin-3	N/A	1	N/A
NNU_09020-RA	Similar to CNB1: Calcineurin subunit B	1	N/A	N/A
NNU_13577-RA	Similar to Caltractin	1	1.42	N/A
NNU_22365-RA	Similar to Calnexin homolog	1	0.73	0.92
NNU_13438-RA	Similar to ANN1: Annexin D1	1	N/A	1.24
NNU_22480-RA	Similar to ANN2: Annexin D2	1	N/A	1
NNU_01039-RA	Similar to ANN3: Annexin D3	1	N/A	N/A
NNU_15418-RA	Similar to ANN4: Annexin D4	1	N/A	N/A
NNU_14855-RA	Similar to ANN5: Annexin D5	1	N/A	N/A
NNU_01040-RA	Similar to Annexin-like protein RJ4	1	1.4	2.65
NNU_15823-RA	Similar to CPK2: Calcium-dependent protein kinase isoform 2	1	N/A	N/A
NNU_03392-RA				
NNU_01413-RA	CPK3: Calcium-dependent protein kinase 3	1	N/A	N/A
NNU_09473-RA	CPK4: Calcium-dependent protein kinase 4	1	N/A	N/A
NNU_04896-RA	CPK11: Calcium-dependent protein kinase 11	N/A	N/A	1
Auxin signaling				
NNU_23405-RA	Similar to Auxin-induced protein PCNT115	1	1.59	1.14
NNU_10627-RA				
NNU_23183-RA				
NNU_23179-RA				
NNU_06219-RA	AXR4: Protein AUXIN RESPONSE 4	N/A	N/A	1
NNU_11159-RA	Similar to Auxin-repressed 12.5 kDa protein	1	N/A	N/A
NNU_21253-RA	AIR12: Auxin-induced in root cultures protein 12	N/A	N/A	1
NNU_02652-RA	Similar to At4g12780: Auxilin-related protein 1	N/A	1	N/A
NNU_07873-RA	Similar to At4g12770: Auxilin-related protein 2	N/A	1	N/A
NNU_14842-RA	Similar to IAR1: IAA-alanine resistance protein 1	N/A	N/A	1
NNU_23868-RA	Similar to SKP1A: SKP1-like protein 1A	1	2.39	1.43
NNU_18445-RA	Similar to SKP1B: SKP1-like protein 1B	N/A	1	N/A
NNU_14125-RA	Similar to CUL1: Cullin-1	N/A	N/A	1
NNU_05447-RA	Similar to RCE1: NEDD8-conjugating enzyme Ubc12	1	1.8	1.39
NNU_16590-RA				
NNU_24561-RA	Similar to CAND1: Cullin-associated NEDD8-dissociated protein 1	1	N/A	3.33
NNU_24562-RA				
NNU_06166-RA	Similar to ECR1: NEDD8-activating enzyme E1 catalytic subunit	1	N/A	N/A
NNU_03382-RA	Similar to AXR1: NEDD8-activating enzyme E1 regulatory subunit	N/A	N/A	1
NNU_04189-RA	ILL1: IAA-amino acid hydrolase ILR1-like 1	1	1	0.69
NNU_25480-RA	ILL7: IAA-amino acid hydrolase ILR1-like 7	N/A	N/A	1
14–3-3-like proteins				
NNU_07652-RA	Similar to 14–3-3-like protein	1	0.52	1.14
NNU_00542-RA				
NNU_22679-RA	Similar to 14–3-3-like protein (*Pisum sativum*)	1	0.4	1
NNU_13400-RA	Similar to TFT7: 14–3-3 protein 7	1	N/A	N/A
NNU_04500-RA	Similar to GRF12: 14–3-3-like protein GF14. Transcription activator that plays a regulatory role in gibberellin-induced stem elongation.	1	1	N/A
NNU_00971-RA	Similar to GF14D: 14–3-3-like protein D	1	N/A	0.5
NNU_08512-RA				
NNU_13288-RA	Similar to GRF8: 14–3-3-like protein GF14 kappa	1	N/A	1.28

^a^The relative expression level was normalized within each protein itself. N/A, no detection

Besides these signaling proteins, some other functional proteins were also enriched. A total of 92 ubiquitin/26S proteasome pathway related proteins were identified in this study (Additional file 2: Table S4). These included 34 proteasomal subunits, 2 E1, 14 E2, 26 E3 and E3 binding proteins, and 7 de-ubiquitination proteins. Although the accumulation of E2 was a little bit complicated, E1 and most of E3 had higher levels at S1 and S3. Eight aquaporins and 61 intracellular trafficking related proteins were identified, and had high level at S1 and S3 (Additional file 2: Table S4). Consistently, a total of 25 small GTPase, which mainly function in signal transduction, cell proliferation, cytoskeletal organization, and intracellular membrane trafficking, were also identified as preferentially accumulating in S1 and S3 (Additional file 2: Table S4).

## Discussion

Formation of temperate lotus rhizome is a very complicated but well-regulated physiological process, which experiences a series of changes at morphological, physiological and biochemical as well as molecular level. The underlying mechanisms of this process are still elusive. Focusing on the process from stolon stage (S1) to middle stage (S2), and then to rhizome stage (S3), we employed comparative proteomic study to identify the potential functional important proteins for rhizome formation and enlargement, which might help to shed light on the mechanism of this process. Previously, we conducted transcriptomic analysis on the same stages of rhizome enlargement [7]. Based on the correlation analysis between the proteomic and transcriptomic data, it seems that they have very poor correlation (Fig. 6d-f), although the correlation for those genes showing similar trends at mRNA and protein levels were high (Fig. 6g-i). Numerous studies have shown that integration of transcriptomic and proteomic analyses could provide more comprehensive insights for the target processes [33–36]. Notably, two studies have shown the regulation of gene expression that occurred at different levels through the combination of transcriptomic and proteomic data [18, 35]. Based on these previous conclusions, it is reasonable to find the limited correlation between our transcriptomic and proteomic data. This inconsistency between mRNA and protein level might either due to the translation efficiency and posttranslational modification or the unsynchronized gene expression at mRNA and protein levels. It might further highlight that transcriptomic and proteomic analyses are both necessary and complementary to each other.

It is known that the initiation of lotus rhizome enlargement occurs in autumn, an observation supported by the morphological data in this study, and indicates the possible involvement of light signaling in this process. In this study, a total of 9 distinct proteins involved in light signaling and 2 in circadian pathways were identified, including the major components COP9, PHYA, FYPP and HY5. The COP9 signalosome is a highly conserved complex, which consists of 8 subunits, and could promote skotomorphogenesis in Arabidopsis [37]. On the contrary, HY5 is a negative regulator of skotomorphogenesis, which is degraded by COP1 under darkness [38]. The expressional patterns of COP9 subunits and HY5 in lotus rhizome indicate that there is an elongation growth at S1, which stops at S2. It is reported that FYPP could interact with and dephosphorylate PHYA, and overexpression of FYPP suppresses flowering [39]. The FRIGIDA protein could also repress flowering through activation the flowering repressor FLC [40]. The accumulation of FYPP and FRIGIDA proteins occurred at S1 and S2, respectively. This implies the inhibition of flowering before the initiation of rhizome, which is consistent with the lotus growth phenomena. In potato, PHYF was proven to be involved in the induction of tuber through forming heterodimers with PHYB [41]. Together, this suggests that light signaling is important for the formation of storage organs in plant. However, different light signaling components might be involved in different plant species.

Additionally, phytohormone related proteins play an important part in formation of storage organs, including auxin, cytokinin, ethylene, abscisic acid (ABA), and gibberellic acid (GA) [10, 42]. Auxin is a crucial regulator in plant growth, including root growth and tuber formation [42–44]. Before and at the initial stage of potato tuber formation, auxin content increases dramatically and then remains at relatively high level, which indicates the function of auxin in the tuber formation [43]. In the present study, 16 auxin related proteins were identified, with most of them showing highest expression at S2 (Table 2), suggesting that these proteins might be involved in the initiation of lotus rhizome enlargement. Among them, 6 are components of the E3 ubiquitin ligase complexes SCFs (abbreviation for SKP1, CDC53 or cullin, F-box protein), which is known being involved in auxin response [45]. This suggests that auxin could induce the cell division and growth, since the cell growth and division related terms were enriched at S2 (Fig. 5b), which could also be supported by the morphological data. It is reported that COP9 could also interact with SCFs, and positively regulate auxin response [46]. The expression of COP9 subunits and the components of SCFs were generally consistent, which indicate that light and auxin signaling might coordinately function to initiate the enlargement of lotus rhizome.

In addition to light and auxin, calcium could also be involved in the regulation of rhizome expansion. Among the 34-calcium signaling related proteins, there were calcium transporters, Calmodulins, annexins and CPKs, which constitute the components in the whole process of calcium signaling. The Ca^2+^ transporters contain the components that regulate both the inter- and intra-cellular Ca^2+^ homeostasis (Table 2). It has been reported that the calmodulin could regulate not only the transporters of other ions [47], but also the aquaporin [48].

Carbohydrate metabolism, including sugar synthesis, transportation, consumption, and storage, plays a critical role in plant life cycle [49, 50]. Sugar metabolism proteins including sucrose synthase and malate dehydrogenase are involved in starch biosynthesis [51], and hence determine the tuber yield in potato [52, 53]. In the present study, sucrose synthase protein (NNU_19077-RA) showed an increase from stage S2 to S3, indicating that sucrose synthase might be involved in the starch accumulation of the lotus enlarged rhizome. Moreover, 14–3-3 proteins are a family of conserved regulatory molecules in all eukaryotic cells, having the ability to bind more than 200 signaling proteins, and are involved in signal transduction and plant development [54–57]. The 14–3-3 proteins played a part in regulating sugar metabolism, and st14–3-3 s were reported to regulate potato tuber formation together with stSP6A and stFDL1 [54]. Similar to sucrose synthase protein, the 14–3-3 proteins in the present study also showed an up-regulated expression from stage S2 to S3, leading to speculation that the 14–3-3 s in lotus function as a platform to recruit other proteins to work on the rhizome formation. As mentioned, rhizome is a storage tissue. This proteomic data was also consistent with the microscopy observation. Altogether, it is very clear that reserves (mainly starches) accumulation occurs along with the enlargement of lotus rhizome.

**Fig. 8:**
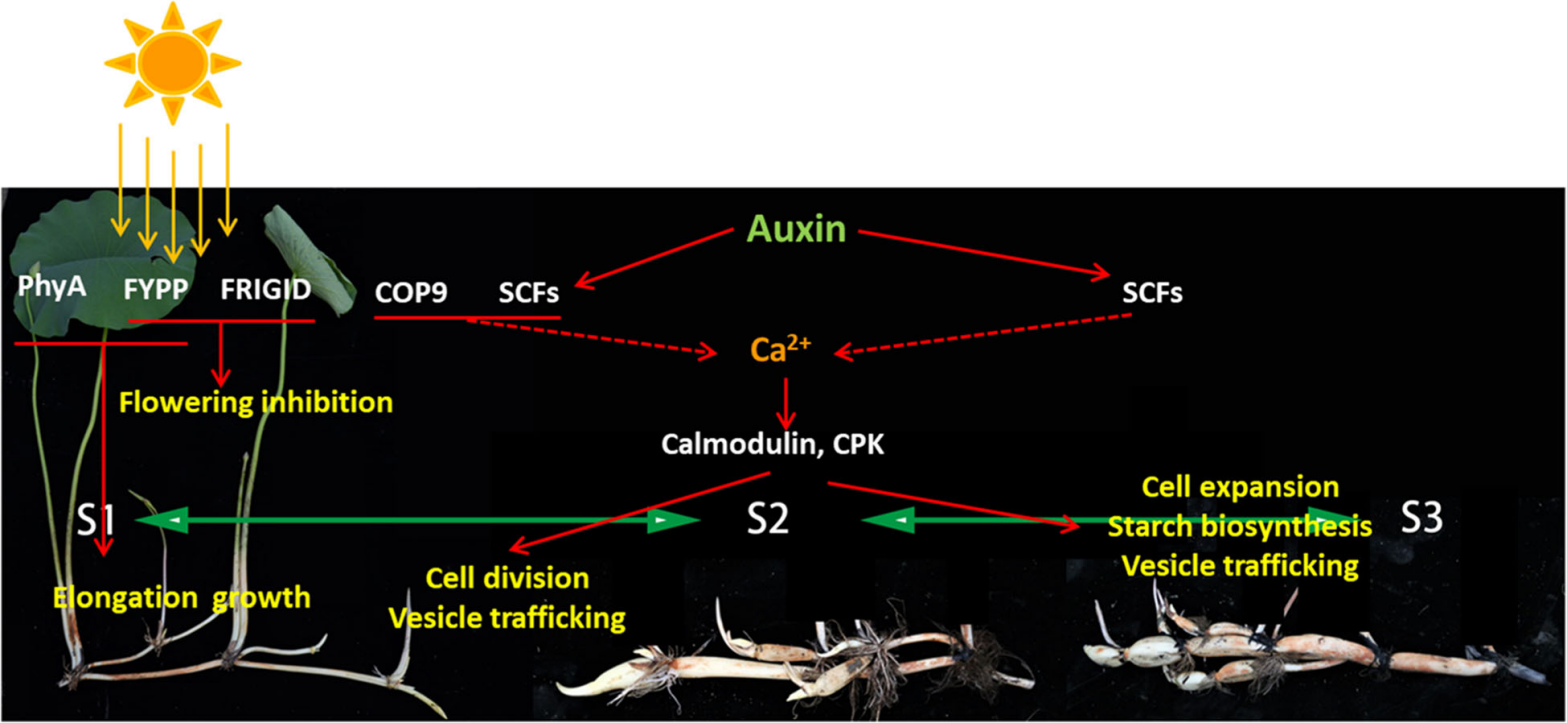
Working model for the initiation of rhizome enlargement in lotus

Based on these results and discussion, we proposed a working model for the lotus to initiate the rhizome enlargement (Fig. 8). Before the second half of August, the rhizome is undergoing the elongation growth. Upon the coming of autumn, the plants sense the changing light signal, and inhibit flowering through the coordinate function of FYPP and FRIGIDA Proteins. Meanwhile, auxin signaling is initiated to promote the cell division. The second messenger Ca^2+^ might also be involved, through which the aquaporin activity and vesicle trafficking are promoted. These signaling could help to enhance not only the expansion of rhizome, but also the biosynthesis of starch.

## Conclusion

In this study, we conducted systematic morphological and proteomic analyses on the process of lotus rhizome enlargement from the stolon stage to the rhizome formation stage. The results showed that lotus rhizome enlargement is mainly caused by the fast occurring cell division and growth, during which the photoperiod and phytohormone play important regulatory roles through secondary messenger Ca^2+^. The cell division mainly happens at the early stage of rhizome development, whereas, cell growth contributes at the S3 stage, during which starch biosynthesis and accumulation are initiated. Based on these, a working model is proposed, which might help to understand the mechanism of lotus rhizome enlargement.

## Supplementary information

**Additional file 1. Table S1.** MS and peptides information for the 2713 identified proteins and the RNA-Seq data for the corresponding genes.

**Additional file 2: Table S2.** Information for the differentially expressed proteins. **Table S3.** KEGG pathway analysis on the differentially expressed proteins. **Table S4.** The functional important proteins.

## Data Availability

The mass spectrometry proteomics data have been deposited to the ProteomeXchange Consortium (http://proteomecentral.proteomexchange.org) via the PRIDE partner repository (33) with the dataset identifier PXD010388.

## Abbreviations

ABA: Abscisic acid
DEPs: Differently expressed proteins
GA: Gibberellic acid
GO: Gene ontology
KEGG: Kyoto Encyclopedia of Genes and Genomes
MS: Mass spectrometry
SSPs: Stage-specific proteins
